# Creative thinking as orchestrated by semantic processing vs. cognitive control brain networks

**DOI:** 10.3389/fnhum.2014.00095

**Published:** 2014-02-24

**Authors:** Anna Abraham

**Affiliations:** Department of Community Medicine and Behavioral Sciences, Faculty of Medicine, Kuwait UniversityJabriya, Kuwait

**Keywords:** creative cognition, divergent thinking, semantic cognition, cognitive control, inhibitory control, fronto-striatal network, fronto-parietal network

## Abstract

Creativity is primarily investigated within the neuroscientific perspective as a unitary construct. While such an approach is beneficial when trying to infer the general picture regarding creativity and brain function, it is insufficient if the objective is to uncover the information processing brain mechanisms by which creativity occurs. As creative thinking emerges through the dynamic interplay between several cognitive processes, assessing the neural correlates of these operations would enable the development and characterization of an information processing framework from which to better understand this complex ability. This article focuses on two aspects of creative cognition that are central to generating original ideas. “Conceptual expansion” refers to the ability to widen one’s conceptual structures to include unusual or novel associations, while “overcoming knowledge constraints” refers to our ability to override the constraining influence imposed by salient or pertinent knowledge when trying to be creative. Neuroimaging and neuropsychological evidence is presented to illustrate how semantic processing and cognitive control networks in the brain differentially modulate these critical facets of creative cognition.

The interest in uncovering the brain mechanisms underlying creative thinking, or the ability to generate original yet relevant responses in a given context (Stein, 1953), has a lengthy scientific history that dates back at least to the 1940s (Reitman, 1947; Ashby and Bassett, 1949). The most influential issues that have guided investigations on creativity and brain function include enhanced creative ability following brain damage (Miller and Miller, 2013), the dominance of right over left hemisphere function in creative thinking (Mihov et al., 2010), and the brain basis of exceptional ability among experts in creative domains such as music, art and dance (Bengtsson et al., 2007). The general (although not necessarily unanimous) picture that emerges from the literature is that creative performance and/or ability is particularly associated with frontal lobe (FL) function (Dietrich and Kanso, 2010), higher right brain activity (Mihov et al., 2010), greater EEG alpha power which reflects high internal processing demands (Fink and Benedek, 2012), and that it can be inadvertently boosted as a consequence of specific types of brain damage (Seeley et al., 2008).

Such generalizations regarding creativity and brain function primarily arise from adopting a somewhat unitary approach in investigating creativity where it is assessed as an undifferentiated general construct, as opposed to process-differentiated one. This is customarily achieved by contrasting brain activity (neuroimaging or EEG studies) or behavioral performance (neuropsychological studies) during creative vs. non-creative tasks. Several researchers have critically addressed theoretical and methodological concerns that arise in the context of neuroscientific investigations of creative thinking, such as the inability to prompt creativity in a reliable or valid manner and the suboptimal nature of comparison tasks in creativity paradigms (Dietrich, 2007; Arden et al., 2010; Sawyer, 2011; Abraham, 2013). The advantage of a unitary approach is that it delivers the “big picture” regarding our creative brains. However, the unitary approach is too generalized, and hence insufficient, if the overarching aim is to uncover the neural and information processing mechanisms by which creativity occurs. As several cognitive operations work in unison when we are engaged in creative idea generation, adopting a “process” approach to creativity (Kozbelt et al., 2010) in investigating the brain correlates of these different operations would allow us to realize such an objective.

## Cognitive components of creativity

The Geneplore model of creativity (Ward et al., 1995; Finke et al., 1996; Ward et al., 1997), which sought to characterize the different mental operations that are involved during creative thinking, was driven by the process approach. Although diverse in nature, these operations were proposed to have two components in common. First, they involve the generation of potential ideas or “preinventive” structures (e.g., the analogical transfer of information from one domain to another). Second, this initial generation phase is followed by extensive exploration of these preinventive structures (e.g., search for conceptual limitations).

**Figure 1:**
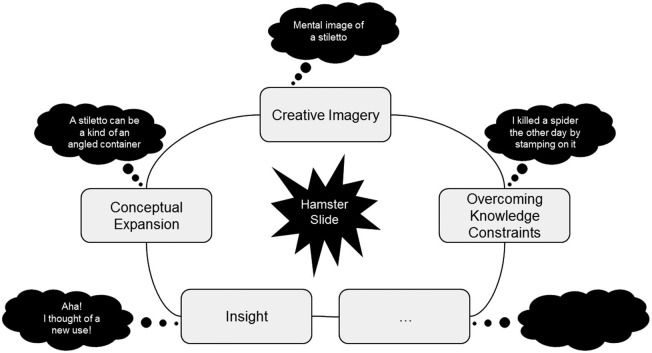
**A schematic diagram to highlight four of the several different mental operations that are involved in creative thinking with a hypothetical example of how different aspects of creative cognition work in unison during creative idea generation**.

According to this model, the essential difference between creative and “non-creative” or normative cognition does not lie in the type of mental operations themselves, but in the contexts to which these information processing toolboxes are applied (Abraham, in press). The contexts or problem solving situations that prompt creative cognition (e.g., compose a haiku) are relatively more open-ended, ambiguous, non-linear, abstract and unpredictable compared to those that primarily necessitate normative cognition (e.g., devise a weekly exercise regime). Creative cognition can therefore be assessed by examining normative cognitive processes under explicitly generative conditions. In fact, a number of such mental operations have been described (Figure 1; Abraham and Windmann, 2007). These include the ability to broaden the framework of established conceptual structures (conceptual expansion), mental visualization during creative idea generation (creative imagery), the ability to surpass the constraining influence of recently activated knowledge (overcoming knowledge constraints), and the sudden occurrence of a solution during problem solving as a result of a fundamental perspective shift (insight). So, how do these operations work in combination with one another during creative idea generation?

Imagine the following scenario. You are asked to imagine new uses for a shoe, beyond the object’s customary use of foot protection. Allow yourself a few minutes to carry out this task before reading further and make note of the uses you generate.

Typically, other common uses for a shoe, such as using it to kill a cockroach, will occur to you automatically. As these familiar options are quickly exhausted, the task becomes increasingly cognitively demanding. While trying to come up with novel ideas, you probably generated mental images to explore a shoe’s physical parameters in terms of weight, volume, dimensions, materials, type: stilettos vs. sneakers, and so on (creative imagery). During this process, it may have become apparent to you that a shoe can be used as a make-shift container (conceptual expansion) and that the brutally angled sole of a stiletto lends itself to different uses than the flat sole of a sneaker. While exploring potential uses for a shoe as a highly angled container in the case of a stiletto, you may inadvertently recollect uses that are closely related to those you have already generated, such as using the stiletto’s heel to impale a spider, and you strive to inhibit this tendency to rehash known associations (overcoming knowledge constraints). Then, seemingly out of the blue, the different elements that are being explored suddenly come together in a novel manner while you undergo some form of an “aha-experience” (insight) as you become aware of this new use of, for instance, using a shoe as a hamster slide (Figure 1).^1^

The focus in the present opinion article will be limited to evaluating the similarities and differences in brain function that are associated with conceptual expansion and overcoming knowledge constraints as these operations have received little to no attention thus far within the literature, unlike the processes of insight (Kounios and Beeman, 2009; Dietrich and Kanso, 2010) and imagery (Farah, 1989; LeBoutillier and Marks, 2003; Bartolomeo, 2008).

## Conceptual expansion

The ability to expand acquired conceptual structures to include novel elements is investigated in tasks that assess conceptual expansion (Ward, 1994). The original task involved having participants generate animals that lived on another planet that was very different from Earth. How far these alien creatures deviated from generic Earth animals in terms of the absence of typical features and the presence of atypical features was assessed. As this animal task cannot be optimally implemented in its original form for neuroimaging research, three alternative experimental paradigms were developed to assess conceptual expansion. These have now been implemented in fMRI (Abraham et al., 2012b; Kröger et al., 2012; Rutter et al., 2012b) and EEG settings (Rutter et al., 2012a; Kröger et al., 2013).

The paradigms were devised with the objective of uncovering the brain correlates of conceptual expansion. Brain regions that were commonly activated across all three paradigms would be considered to be reliably involved in creative conceptual expansion. Two approaches were adopted when developing these paradigms^2^ where one was devised to assess “active” conceptual expansion (Abraham et al., 2012b) while the other assessed “passive” conceptual expansion (Kröger et al., 2012; Rutter et al., 2012b). Participants shouldered the task of expanding the concepts themselves (generate novel uses for a newspaper) in the volitionally generated or active conceptual expansion paradigm. In contrast, during the involuntarily induced or passive conceptual expansion paradigms, participants were presented with object-use combinations (Shoe → Plant pot) or metaphors (The clouds danced over the city), to which they reported experiences of conceptual expansion. This occurred when they encountered an object-use combination or metaphor that was deemed by them to be both novel (previously unknown to them) and appropriate.

The brain regions that were found to be activated across all three paradigms were limited to the left hemisphere and included the anterior inferior frontal gyrus (IFG: BA 45/47), the temporal pole (TP: BA 38) and the lateral frontopolar cortex (FPC: BA 10). The IFG is central to semantic information processing in the brain (Bookheimer, 2002; Binder and Desai, 2011; Jefferies, 2013) with anterior aspects of this structure being involved in semantic selection and controlled semantic retrieval (Thompson-Schill, 2003; Badre and Wagner, 2007). The TP is also key structure of relevance in semantic cognition as it is widely held to underlie the domain-general or amodal repositories of conceptual knowledge of the brain (Lambon Ralph et al., 2009; Simmons and Martin, 2009). It is involved in the combination and integration of lexical representations to a context (Lau et al., 2008), as well as in the acquisition of new conceptual knowledge (Hoffman et al., 2014).

The lateral FPC, in contrast, is held to mediate cognitive control at the most abstract level of information processing (Badre, 2008; Christoff et al., 2009) and plays a key role during relational reasoning (Christoff et al., 2001; Wendelken et al., 2008) as well as when combining information from two or more separate cognitive operations (Ramnani and Owen, 2004). Although the lateral FPC is not specifically limited to semantic aspects of information processing, both this brain region and the anterior IFG are sensitive to the degree of associative strength between concepts (Bunge et al., 2005; Green et al., 2010) with greater brain activity elicited by wider semantic distances.

To summarize, neuroimaging studies on conceptual expansion have revealed that brain structures (inferior frontal, temporopolar and frontopolar) that are collectively associated with the selection, controlled retrieval, combination and integration of semantic knowledge are preferentially more strongly engaged during creative conceptual expansion relative to other types of normative semantic information processing. Although nonverbal conceptual expansion has yet to be investigated in the same manner, the same brain network would be expected to be involved in conceptual expansion regardless of the stimulus type. This is because the use of verbal and non-verbal as well as semantic and non-semantic control tasks across the different paradigms revealed that the engagement of this network of brain regions during conceptual expansion cannot be merely attributed to verbal or semantic processing.

## Overcoming knowledge constraints

The ability to override the hindering influence imposed by relevant but distracting information during creative idea generation (Smith et al., 1993) is referred to here as the process of overcoming knowledge constraints. In the original toy task that was devised to assess this operation, participants are asked to imagine and draw a novel toy that does not yet exist (previously/currently). But before they do so, they are shown examples of three novel toys that were generated by others. Unbeknownst to the participants, these novel toy examples were in actuality engineered by the experimenter to have three features in common. What is assessed after the participants generate their own responses is how many of these three elements are present in the participants’ toy inventions. Higher scores reflect stronger incorporation of example elements, which in turn reflects a poorer ability to overcome knowledge constraints that were levied by a salient and distracting context.

Just as in the conceptual expansion task, the toy task cannot be optimally implemented in its original form for neuroimaging research. To date, no study has directly investigated the brain correlates of overcoming knowledge constraints during creative idea generation. Only two neuroimaging studies have an indirect bearing on this discussion where the opposite effect, of “cognitive stimulation” on creative problem solving upon being exposed to others’ ideas, was assessed (Fink et al., 2010, 2012).

The behavioral findings indicated that prior exposure to common uses (generated by others) relative to no prior exposure, led to greater originality in self-generated uses. But this was not true with prior exposure to original uses. As Fink et al. (2012) did not assess the degree of similarity between self-generated vs. other-generated uses, it is not possible to speculate about how these two situations may have involved overcoming different types of knowledge constraints. When comparing the brain’s response during idea generation following original-use-prior-exposure compared to no-prior-exposure or common-use-prior-exposure, heightened activity was found in the left posterior middle temporal gyrus (MTG). The posterior MTG is part of the brain’s semantic system (Binder and Desai, 2011) and is held to underlie the “long-term storage of and access to information associated with lexical representations” which “serves as input to higher-order semantic processes” (Lau et al., 2008). If we were to presume that prior exposure to original ideas imposes more constraints on idea generation than no prior exposure to ideas, as has been suggested by behavioral research, one could postulate that posterior middle temporal regions are more actively recruited when having to overcome knowledge constraints.

Interestingly, neuropsychological evidence has demonstrated that damage to lateral parietal and temporal cortices (including the posterior MTG) is associated with poorer performance on the overcoming knowledge constraints toy task (Abraham et al., 2012a). Creative cognition was assessed in three neurological samples with lesions of the FL, basal ganglia (BG), or parietal-temporal lobe (PTL). The PTL group were significantly less adept at overcoming knowledge constraints, which is a pattern that fits with findings of semantic perseverative responses associated with this population, especially in the presence of semantic distractions (e.g., Corbett et al., 2011).

The BG and FL-POL (FL group with frontopolar/frontoorbital lesions) groups though were found to be better at overcoming knowledge constraints during creative idea generation compared to healthy control groups. This information processing advantage was very specific in that neither the BG group nor the FL-POL group displayed superior performance on any other aspect of creative cognition.

The BG together with the prefrontal cortex are part of the network in the brain that orchestrates executive function and cognitive control (Alexander et al., 1991; Robbins, 2007; Brocki et al., 2008). Within the prefrontal cortex, frontopolar regions underlie abstract cognitive control (Badre, 2008; Badre et al., 2009) while frontoorbital regions are associated with cognitive disinhibition (Cummings, 1993). BG lesions are accompanied by poor inhibitory control, marked inattention and increased distractibility (Fielding et al., 2006; Aron et al., 2007).

These factors would be advantageous in overcoming knowledge constraints as optimal performance on this task requires inhibiting salient information that is engineered such that increased effort must be expended to see past it. Having poor inhibitory control or being easily distractible would render one more capable of overcoming such constraints as one’s attention is continually being involuntarily diverted away from any particular focus.

Further indirect support from this idea comes from a study on creative cognition in attention-deficit/hyperactivity disorder (ADHD) where adolescents with ADHD outperformed healthy matched control participants on the toy task (Abraham et al., 2006). Even within a sample of adults with chronic schizophrenia, a high degree of thought disorder symptoms (disorganization within the semantic content of thought) was associated with superior ability to overcome knowledge constraints on the toy task (Abraham et al., 2007). Indeed, both ADHD and schizophrenia are associated with dysfunctions of the fronto-striatal network in the brain (Robbins, 1990; Bradshaw and Sheppard, 2000; Robbins et al., 2012).

## Creative cognition and the brain

The general picture that glimmers through when bringing together the findings from neuroscientific investigations of conceptual expansion and overcoming knowledge constraints during creative idea generation is that of a dynamic interplay between semantic processing and cognitive control networks in the brain.

Trying to conceive of an original idea necessarily involves broadening or expanding existing conceptual structures to include novel or previously unassociated features. When engaged in conceptual expansion, the brain’s semantic processing network operates on overdrive, particularly the higher-order regions which mediate lexical selection, controlled retrieval, combination and integration processes. During this process of cogitation, when distracting but salient information threatens to throw a spanner in the works by hampering one’s ability to generate truly original ideas, the cognitive control network of the brain storms into play to push these distractions out of one’s mind. This can be done in one of two ways—by either inhibiting or ignoring this salient information.

Inhibiting or ignoring salient task-relevant information is in fact very difficult as our predictive brains are developed to be especially adept at efficient and effective goal-directed action (Bubic et al., 2010) and we are accustomed to operating in our daily lives within normative contexts where the distractions one may have to overcome can be unmistakably recognized and are not necessarily salient or relevant to the specific task at hand. Such distractions can therefore be (relatively speaking) easily ignored. During creative idea generation though, the distracting information can be exceedingly pertinent to the task at hand and cannot therefore go unheeded in the same manner. Under such conditions, imbalances within the fronto-striatal network seem to confer specific advantages in creative cognition, possibly owing to the manifestation of cognitive disinhibition and increased distractibility, which would allow for a greater ease in disregarding salient semantic distractors.

## Conclusions

The objective of this article was to outline the potential neurocognitive mechanisms that underlie two vital aspects of creative cognition—conceptual expansion and overcoming knowledge constraints—based on neuroscientific findings that adopted a process approach to investigate the same. What was highlighted was the role of the semantic processing and cognitive control networks in the brain during creative idea generation. These insights can help inform and guide future neuroscientific investigations on creativity as well as aid in the development of more detailed and targeted information processing models of creative neurocognition. Promising future directions for exploration include the impact of training-induced plasticity effects on different aspects of creative neurocognition as well as uncovering the association between information processing biases in creative cognition with reference to variability that is manifest in real world creativity.

## Conflict of interest statement

The author declares that the research was conducted in the absence of any commercial or financial relationships that could be construed as a potential conflict of interest.

## Footnotes

^1^This example is only presented for illustrative purposes to showcase differences between select cognitive operations during the creative act. As such, it not to be construed as a prototype that represents the manner in which the creative process typically unfolds. The list of selected operations is by no means exhaustive. Moreover, the occurrence, temporal order and interrelation between these operations would be expected to differ as a function of intraindividual, interindividual and contextual factors.

^2^The alternate uses task is widely employed in investigations of creativity where divergent thinking is assessed in terms of fluency or originality of the responses. Just as in studies of other facets of cognition, the same task can be implemented in different ways depending on the microlevel of study to allow for specific inferences to be made regarding creative function. Within the neurocognitive approach, the microlevel is centered on the brain correlates of the cognitive process. So the brain response when performing the alternate uses task is assessed relative to brain response when performing a closely matched control task that does not necessitate conceptual expansion, but nonetheless requires reasoning, fluency, semantic judgments, cognitive control, and so on.
